# Subsequent Use of a Pressure Sensor to Record Intra-Abdominal Pressure After Maximum Vaginal Closure Force in a Clinical Trial

**DOI:** 10.1109/JTEHM.2019.2952245

**Published:** 2019-11-15

**Authors:** Stefan Niederauer, Brian Cottle, Xiaoming Sheng, James Ashton-Miller, John Delancey, Robert Hitchcock

**Affiliations:** 1 Department of BioengineeringThe University of Utah 7060 Salt Lake CityUT84112USA; 2 Department of PediatricsSchool of MedicineThe University of Utah 7060 Salt Lake CityUT84112USA; 3 Department of Mechanical EngineeringUniversity of Michigan 1259 Ann ArborMI48109USA; 4 Department of Obstetrics and GynecologyUniversity of Michigan 1259 Ann ArborMI48109USA

**Keywords:** Pelvic floor disorder, vaginal dynamometer, force measuring speculum, vaginal closure force

## Abstract

Pelvic floor disorders are caused by weakening or damage to the tissues lining the bottom of the abdominal cavity. These disorders affect nearly 1 in every 4 women in the United States and symptoms that drastically diminish a patient’s quality of life. Vaginal closure force is a good measure of pelvic health, but current vaginal dynamometers were not designed for the rigors of hospital reprocessing, often failing due to sensor degradation through repeated sterilization processes. In order to obtain measurements of vaginal closure force in a large study, we designed a vaginal dynamometer that utilizes a removable intra-abdominal sensor already in production for the study. The sensor’s existing data acquisition system was modified to transmit to a tablet allowing the user to view data in real-time. The new speculum design allowed a single sensor to measure vaginal closure force before being used to collect intra-abdominal pressure data in the same study visit. The measurements taken with the new speculum were similar to measurements taken with a previously reported vaginal dynamometer.

## Graphical abstract

We present the design and validation of a real-time, accurate vaginal dynamometer that utilizes a removable intra-abdominal sensor already in production. The new speculum design allows a single sensor to measure vaginal closure force before being used to collect intra-abdominal pressure data in the same study visit.

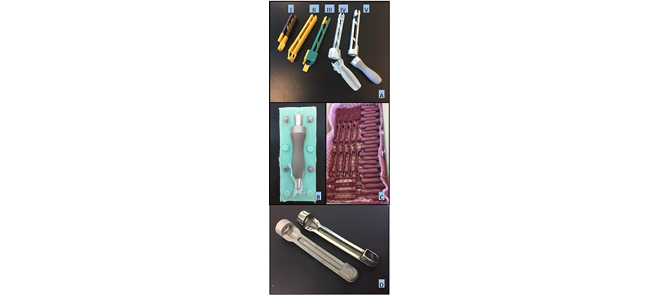

## I. Introduction

Pelvic Floor Disorders (PFDs) affect nearly one in four women in the United States [1]. These disorders are caused by damage and weakening of the pelvic floor muscles (PFM) that close the bottom of the abdominal cavity, and can lead to urinary incontinence, fecal incontinence, and pelvic organ prolapse. The most influential of these muscles are the levator ani muscles. These bilaterally symmetric muscles surround and close the urogenital hiatus through which the urethra and vagina pass. This layer supports the pelvic and abdominal organs [2]. The inability of pelvic floor muscles to hold the hiatus closed can lead to pelvic floor symptoms, primarily pelvic organ prolapse [3].

Vaginal closure force, the force generated by the levator ani muscles adjacent to the distal vagina during a Kegel contraction, is an important measure of pelvic floor function. Maximum voluntary vaginal closure force is the most direct method of evaluating levator ani muscle strength and gives clinicians an objective measurement that can be used to track pelvic floor disease progression and pelvic muscle health. Clinicians traditionally use tests such as the Brinks test or Oxford scale which rely on digital palpation to evaluate the strength of pelvic floor muscles. Although digital palpation is popular because it is inexpensive and simple, it is a subjective measure that has been shown to have poor reliability for pelvic floor muscle strength evaluation [4], [5].

Devices such as balloon perineometers or manometers have been used to assess levator ani strength by changes in intravaginal closure pressure. These pressure measuring devices are often balloon type devices and have significant compliance when under load. Unfortunately, the balloon compliance results in non-isometric levator contractions because the muscle can shorten when contracted and this can introduce significant measurement bias due to the well-known muscle length-tension relationship [6].

**FIGURE 1.**
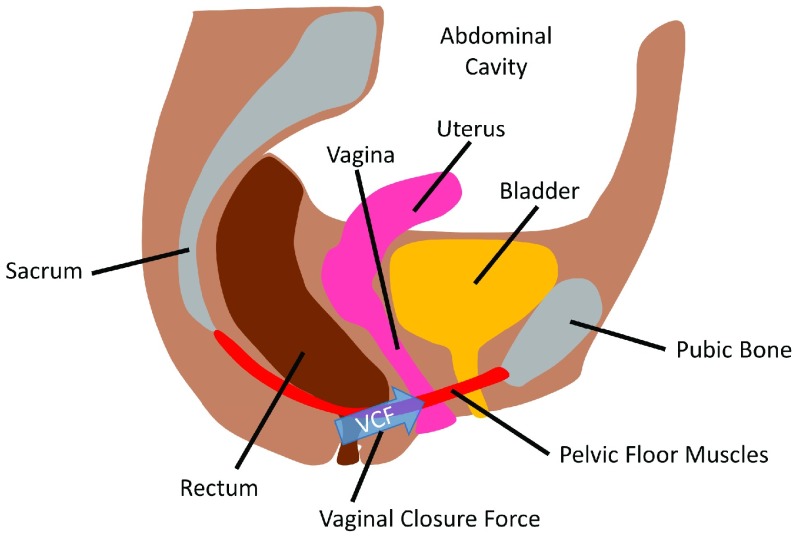
Mid-sagittal plane cross-section of the pelvic floor. Vaginal closure force is measured as the force produced by the pelvic floor muscles in the mid-sagittal plane toward the pubic bone.

To circumvent this problem other devices have been developed to instead measure vaginal contraction force directly, shown in Figure 1, in an isometric fashion [6]–[14]. Such devices have shown a good ability to detect levator ani muscle weakness [3], but many of these devices were designed for small-scale clinical use and are not designed for standard hospital reprocessing for repeated sterile use.

Recent cases of patient cross-contamination from reprocessed medical devices, such as duodenoscopes, has led to increased vigilance on the side of the FDA, The Joint Commission, healthcare facilities, and Institutional Review Boards [15]. Devices undergoing reprocessing are subjected to extreme environmental conditions, such as heat, humidity, and chemicals. Force and pressure sensing transducers incorporated into various vaginal force measuring devices have typically not been designed to withstand this type of sterile reprocessing.

The Motherhood and Pelvic Health (MAP) Study, being conducted at the University of Utah, is collecting data on primiparous women from the third trimester to one-year post-partum [16]. As part of the study, pelvic floor muscle force is evaluated one year postpartum using a reusable vaginal speculum [6] referred to as the ‘fixed-bill speculum’ in this paper. This device performed well during initial participant measurements, but the device’s performance started to decline after the device went through several ethylene oxide (ETO) sterilization cycles. The fixed-bill device was not designed to undergo repeated sterilization cycles, and we hypothesized that the humidity and heat present in the ETO sterilization cycles compromised the adhesion of the strain gauges used on the fixed-bill speculum. After reviewing the design of the fixed bill speculum we determined that the device would likely not survive the repeated use required by our study. In addition, the MAP study required that PFM measurements be taken in over 500 women and the project was not budgeted for single-use devices to determine PFM strength. To accommodate our need to measure PFM strength in numerous participants, we began examining alternative ways to obtain these measurements. One of the goals of the MAP study was to measure intra-abdominal pressure (IAP) in women during various physical activities. To measure IAP, each participant received a sterile, single-use Intra-Vaginal Transducer (IVT) [17]. This IVT device is designed to measure IAP when placed in the upper vagina and utilizes an incompressible silicone gel encapsulated in a silicone elastomer to transfer pressure applied to the elastomeric capsule to a pressure sensor located within the capsule. We hypothesized that the IVT could serve a dual purpose to both measure IAP as well as vaginal closure force by coupling the IVT with a custom speculum, which would allow collection of both vaginal closure force and intra-abdominal pressure using the same sensor. The sensor would first be used in the speculum to collect vaginal closure force data. After measuring vaginal closure force, the IVT would be removed from the speculum and inserted into the upper vagina to monitor intra-abdominal pressure. The custom speculum would then be reprocessed using standard hospital methods and the IVT is stripped of patient-contacting materials and reworked in the lab. We held a preliminary design review meeting with our engineering team, study coordinators, and OBGYN investigators. As a result of this meeting, we determined the requirements of a new force measuring speculum design utilizing the IVT as the sensing element and decided to move forward with the project.

## II. Methods

**FIGURE 2.**
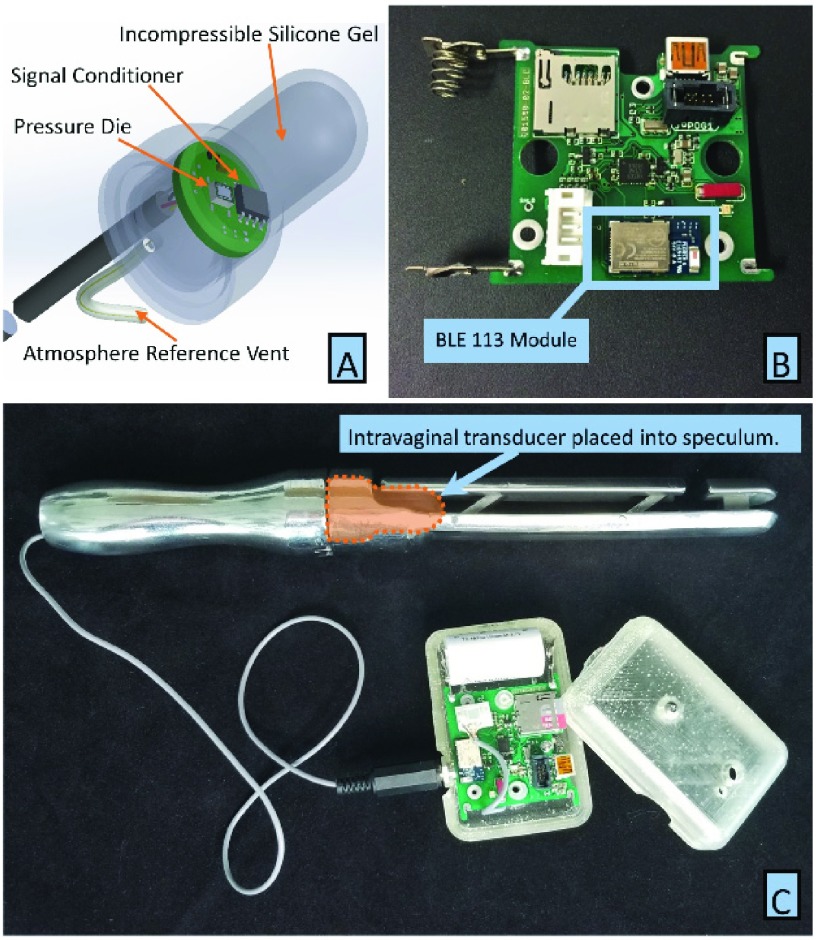
(A) Intravaginal Transducer Assembly and (B) Instrumentation Module circuit board with added BLE 113 Bluetooth Module. A rechargeable battery is placed in the contacts on the left side of the board, which can hold enough charge for approximately ten hours of continual use. (C) Intravaginal Transducer and Instrumentation Module combined with parallel bar speculum to measure vaginal closure force. Forces placed on the distal end of the upper dynamic bill are transferred to IVT at the proximal end of the upper bill and is measured as pressure by the IVT.

The redesigned speculum was modeled after the fixed-bill speculum and included a four-bar linkage to transfer the force from the levator ani muscles to the IVT (Figure 2). We chose the four-bar linkage because other designs, such as the shear beam principle used by the fixed-bill device, would not allow enough displacement at the IVT contact point to resolve fine changes in applied force. The four-bar linkage design utilized a static lower bill and a dynamic upper bill arranged parallel to one another and connected by parallel linkage bars. The system consists of the reusable Instrumentation Module (IM), reusable speculum and single use IVT.

**FIGURE 3.**
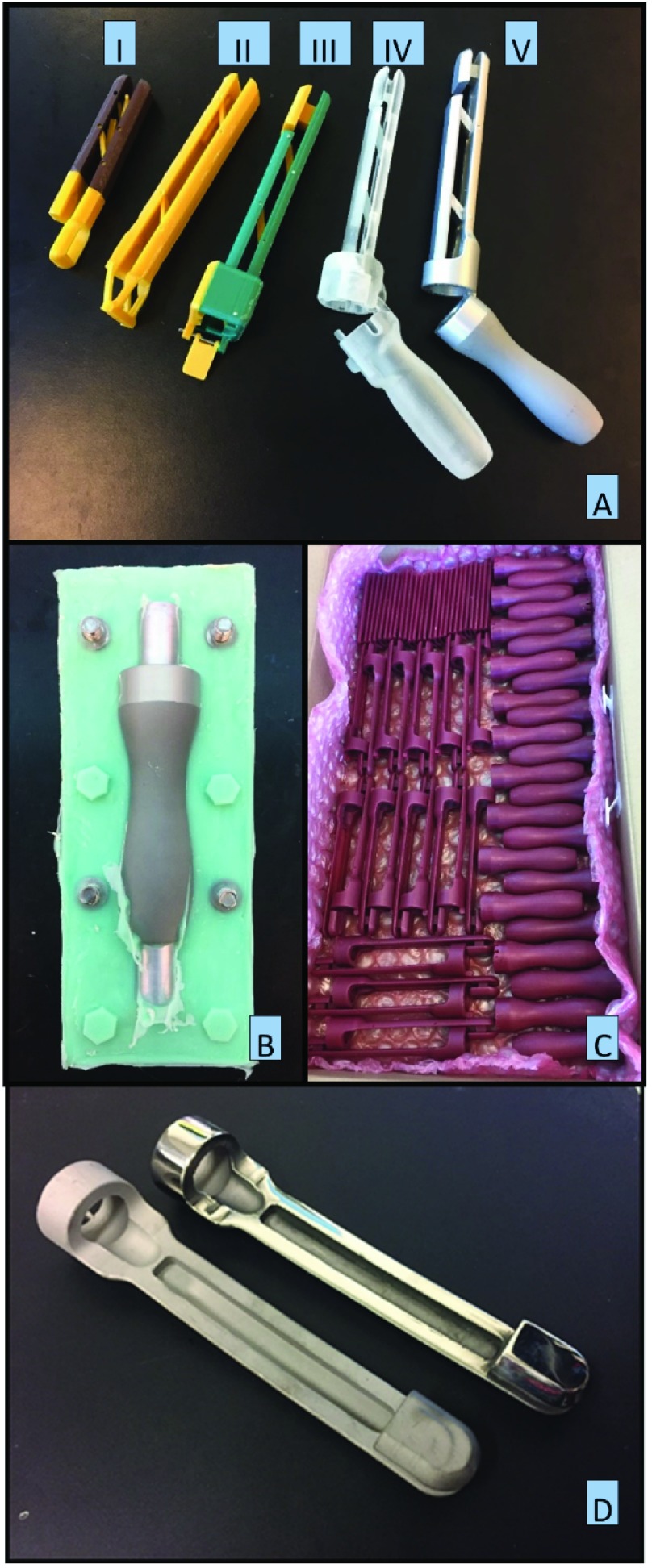
(A) Initial prototype speculums were printed from ABS as seen in (I) (II) and (III). Once a final design was established the speculum was printed from biocompatible MED610 polymer (IV) for preliminary testing. The final prototype was machined from aluminum (V) for further testing and to act as a master for the investment casting. (B) Silicone molds were made from the machined aluminum master parts. Shown here is the speculum handle with the core plug. (C) The molds allowed for rapid reproduction of each speculum component in wax for subsequent investment casting using stainless steel. (D) Investment cast stainless steel components were cast from each wax part. The as-cast parts (left) were polished (right) prior to final assembly.

Proof-of-Concept prototypes were printed using a 3D printer (Ultimaker 3, Dynamism) with ABS material (Figure 3A). In the first prototypes, we experimented with the IVT-speculum contact interface, number of linkage bars (2 or 3), and linkage bar location and size. We found that the IVT responded linearly to weights hung at the distal end of the dynamic bill. We also determined that there was no difference between 2 or 3 linkage bar designs and that linkage bars should be placed as far as possible from one another to better maintain parallelism between the fixed and dynamic bills.

Based on the proof-of-concept prototype, a more detailed design was developed that included an interface surface that is the negative of the IVT boss and fillet shapes, a pivoting and locking handle to allow for IVT placement, and a static bill end to remove IAP crosstalk as described by Ashton-Miller *et al.* [6]. To keep the most prominent and effective attributes of the improved instrumented fixed-bill speculum we designed our speculum to mimic the dimensions and properties of this previously described speculum. The overall shape of the two designs is similar and is designed to be held and used with one hand. The dimensions of the patient contacting portion of the bills (distance between the outer surfaces of the bills, width of the bills and distance between the bills) vary by no more than 2 mm from the fixed-bill speculum dimensions. Our removable sensor speculum is longer overall (28.2 cm vs. 16.7 cm) to include space for housing the IVT as well as a handle for ease of use. The bills are 23.6 mm wide and the external surfaces of the top and bottom bills are separated by 23.4 mm. The static bill at the distal end of the speculum is 29.6 mm long and the dynamic measuring bill is 116.8 mm long.

The first functional prototype (Figure 3A) was printed out of a biocompatible photo-cure polymer (MED610, Stratasys). This polymer can be steam sterilized and has been validated for contact with mucus membranes for up to 24 hours [18]. This functional prototype was provided for user validation by having the study coordinators who would use the device in the clinic use the device in a mock visit and provide feedback on function and feel. The coordinators provided recommendations including larger radius fillets on all surfaces to improve the appearance and feel of the device for the study participants. During laboratory bench testing of the photo-cure polymer speculum, it became apparent that the photo-cure polymer would not be suitable for the intensive use anticipated for our clinical study. Concerns of polymer degradation from sterilization cycles, the polymer’s porosity, and the overall brittle nature of the polymer created safety concerns that our design team concluded was unsuitable for our application. The design team decided to pursue the same design out of 316 stainless steel, a common material for surgical instruments and speculums that has a successful track record of sterile reprocessing.

Before transitioning the material from the MED 610 polymer to stainless steel, an aluminum speculum was machined from 6061-T6 and underwent a clear anodization process (Figure 3A). This final design used three machined parts: a dynamic upper bill, a static lower bill with supporting tip, and a handle that hinged at the proximal end of the lower bill. The upper and lower bill had holes drilled laterally through the pieces for press-fit pins that would act as pivot points for the linkage bars. Two linkage bars were plasma cut from 10.15 mm thick aluminum plate and reamed to slip-fit tolerance of the pins placed through the upper and lower bills. The handle and lower bill were fitted with press-fit neodymium magnets (M116$\times$14CYL, Apex Magnets) that held the handle closed after the IVT was placed in the speculum. Bench testing confirmed that the aluminum device met the design requirements of response linearity and repeatability accurate to within 5% of full scale (20 N).The machined aluminum parts were used as “masters” in order to investment cast the desired number of speculums from stainless steel.

Silicone molds were created using the machined aluminum parts (Figure 3B). The dynamic upper bill used a simple two-part mold, but both the handle and static lower bill required that mold cores be incorporated to reduce post-casting machining operations. Microcrystaline molding wax (KC 1467-A, Paramelt) was melted in a water bath and poured into these molds, which were pre-heated to reduce chances of premature wax solidification in the thin-wall passages of the mold. The cast waxes were removed after solidification and inspected for defects. Approximately 20 wax parts were made of each component and sent to a local foundry for investment casting with 316 stainless steel (Intermountain Precision Casting; Lindon, UT).

After receiving the cast stainless parts, those parts with thin-wall defects were rejected before the remaining pieces were polished (Figure 3D) and final machining operations performed for placement of press-fit pins and magnets. The linkage bars were water-jet cut from 10.15 mm thick 316 stainless steel plate and reamed for slip-fit tolerance of the press-fit pins that would be placed through the upper and lower bills. All pins and magnets were press fit in place. All pins were ground and polished flat to the exterior surface of the speculum bills.

During use, the speculum data must be visualized so that nurses can correctly place the speculum within the vagina. The placement process ensures that the distal end of the upper dynamic bill is at the urogenital hiatus [6]. This placement eliminates cross talk from intra-abdominal pressure and increases measurement accuracy. The IVT is connected by a small cable to the IM which supplies power and stores data (See Figure 2C). To reduce the design cycle time of incorporating live data streaming, we decided to incorporate a stand-alone Bluetooth Low Energy (BLE) module (BLE113, Bluegiga). This module was integrated onto the IM board where space was available (Figure 2B). The master microcontroller on the IM was programmed to send IVT measurement data to the BLE module using UART protocol, and the BLE module was programmed to sense new UART data and update the Bluetooth GATT profile so that another wireless device could display the data.

An Android-based tablet (NEXUS 7, ASUS) was used as the data display for this system. A companion app for the speculum system was created using App Inventor, a GUI-based online programming application for Android systems. The companion app for the removable speculum device received raw pressure data from the IVT through a Bluetooth-Low Energy connection with the IM at the IVT measurement rate of 32 Hz. The after establishing a Bluetooth connection the user takes an in-air measurement and a calibration measurement, which the companion app then uses to convert subsequent measurements received from the IVT to force measurements using a transfer function described later. Data is plotted on the app in real time to assist user placement of the speculum using the same method as with the fixed sensor speculum. To place the speculum correctly, the speculum is first inserted “too far”, then the participant strains to produce IAP without contracting the pelvic muscles.

At the same time, the speculum is slowly withdrawn until the influence of IAP is no longer present in the tracing, but pelvic muscle contraction still registers [6]. The app was designed to provide visual feedback to the user for correct speculum placement, as well as streamline data collection for the MAP study, with single-button wireless connection to the IM, automatic calculation of maximal force for each activity, and activity prompts to direct study personnel through each visit. After collecting the raw data, the app calculated the single maximum point for each ten second activity window, which was time-marked with a wireless Bluetooth foot pedal (PEDpro, AirTurn) or by pressing the “activity mark” button during data collection. The kegel rep activity was calculated as a mean maximum for the three repetitions. All visit data, including calibration and raw IVT data, were stored locally on the tablet for data quality and integrity checks.

All stainless speculums were tested in the laboratory at room temperature by inserting an IVT into the speculum, clamping the speculum around the hinge point with a 3 prong chemistry clamp, and hanging brass weights from the distal end of the upper dynamic bill. All IVTs used in bench testing were pressure calibrated for clinical use before being placed in the speculum. The IVT sensor signal conditioner (ZSC31014, IDT) reports pressure as a 14-bit number for values from 0 to 5 psig (i.e., 0 psig is 0 binary and 5 psig is 16323 binary). The reported pressure measurements along with known applied forces from the hanging weight were used to develop a pressure-force transfer function.

During bench-testing of the speculum with several IVTs, the slope and offset of the response was noted to vary, but the response was always linear. To improve the accuracy of the speculum, the devices were set up to be calibrated individually and immediately prior to use in the clinic. To calibrate each IVT/speculum pair, a 4 in. spring clamp with known closure force of 10.4 N was placed at a standardized location 2 mm from the distal tip of the upper dynamic bill for each pair. The app directs the user to measure the response of the IVT in the speculum when “in air” and with the calibration clamp applied. These two measurements allowed the companion app to correct for variations in IVT, speculum and IVT placement with a simple linear transfer function unique to each IVT/speculum pair. The transfer function has the form:

\begin{equation*} F_{Speculum}= \frac {\left ({P_{Calibration}-P_{Air} }\right)}{10.4 \frac {PSIG}{N}}\ast P_{IVT}-P_{Air}\end{equation*}

where, $P_{Calibration}$ is the pressure measured by the IVT when the calibration clamp is applied, $P_{Air}$ is the pressure measured when the IVT is placed in the speculum with no force applied, and $P_{IVT}$ is the pressure measured by the IVT when placed in the speculum.

Linearity of the speculum design when coupled with various IVTs was tested by conducting an inter/intra device benchtop study. In this study, 5 IVTs were tested with 5 different stainless steel speculums after calibration with the spring clamp. The speculums were attached to a static fixture and weights were hung from the active bill in the same manner as the linearity testing.

The final speculum system, including the companion app software, was verified by running through the companion app program with several different clinic-ready IVTs. The speculum was set up in the static fixture as before, calibrated with the spring clamp, and weights were hung from the speculum during event recordings in the app. The software-reported values of each activity were then compared with the actual forces applied by the weights.

Statistical analyses used included descriptive statistics, the use of linear correlation coefficients to calculate the linear association for each pairing, and a two-sided Students t-test in which a p value less than 0.05 was considered significant.

The removable sensor speculum was approved for use in the ongoing MAP clinical study by the University of Utah IRB and data were collected from the same study population pool as the fixed sensor speculum. Participants in the study were asked to perform 3 activities: Valsalva, maximal PFM contraction (called a Kegel Hold in this study), and rapid PFM contractions (called a Kegel Repetition in this study) maneuvers with rests in between each activity [16].

## III. Results

**FIGURE 4.**
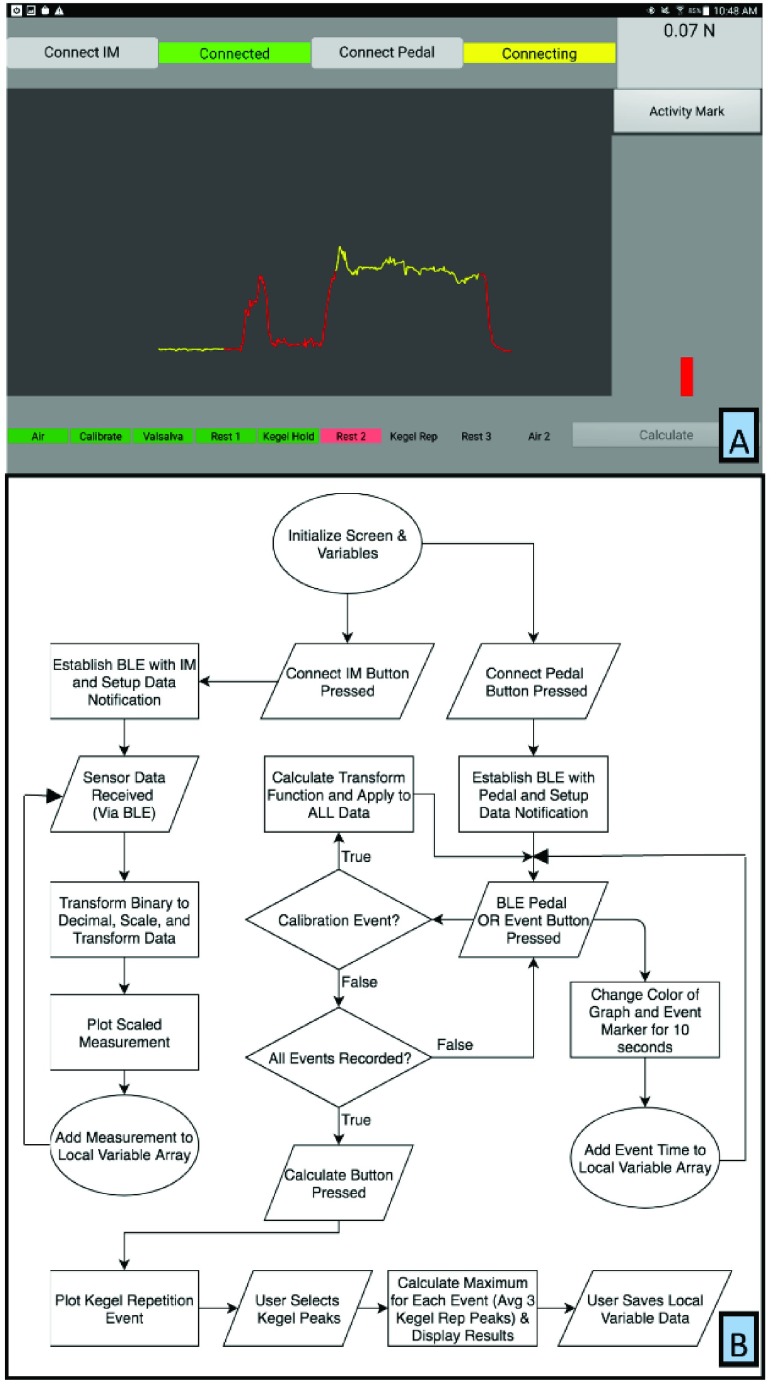
(A) Screen layout of the companion app for the speculum. (B) Companion App signal processing flowchart. The “Connect” buttons at the top of the screen establish Bluetooth connections to either the Instrumentation Module (which initiates data streaming from the speculum) or the Remote Pedal (which marks the beginning of activities). The Force Graph displays force in real-time and changes color from red to yellow when actively recording an activity. The Force Readout and Force Magnitude bars show the live measurement. When a recording session is initiated all of the Event Labels are greyed out (inactive). The Event Labels then turn red (ready for measurement) as the participant protocol progresses. They then turn yellow when actively recording an event. The recording is initiated using the foot pedal switch and then continues for 10 seconds. The Event Labels then turn green when event recording is complete. The app automatically runs through the events from left to right and after all events are recorded, the calculate button becomes available (shown unavailable). After selecting the calculate button, the tracing for the kegel rep activity is plotted and the user selects the three repetitions for analysis. Once the repetitions have been selected the calculate button is pressed again and the results of the visit are calculated and displayed for the user.

The companion app layout (Figure 4) has a large graph area in the middle of layout that continually updates the data received from the IM. Buttons for establishing BLE connections with the sensor and a wireless pedal are at the top left, which are color-coded for the wireless connection state (red for not connected, yellow when establishing a connection, and green during an established connection). A force readout pane and magnitude bar are located on the right to give coordinators a reference for the current force values. Activity prompts are shown at the bottom and are also color coded for ease of use (red when ready to start activity, yellow during measurement window, green when measurement is complete, and greyed-out when inactive). The calculate button at the bottom right graphs the Kegel repetition activity and prompts the coordinators to select the three peaks in the measurement window. After selection, the app automatically calculates the maximum single point for each activity and the mean maximum of the three selected Kegel repetition peaks. These values are then reported on screen for coordinators to record. All data are saved after the coordinator enters the participant number and selects the save button that appears below the reported values.

**FIGURE 5.**
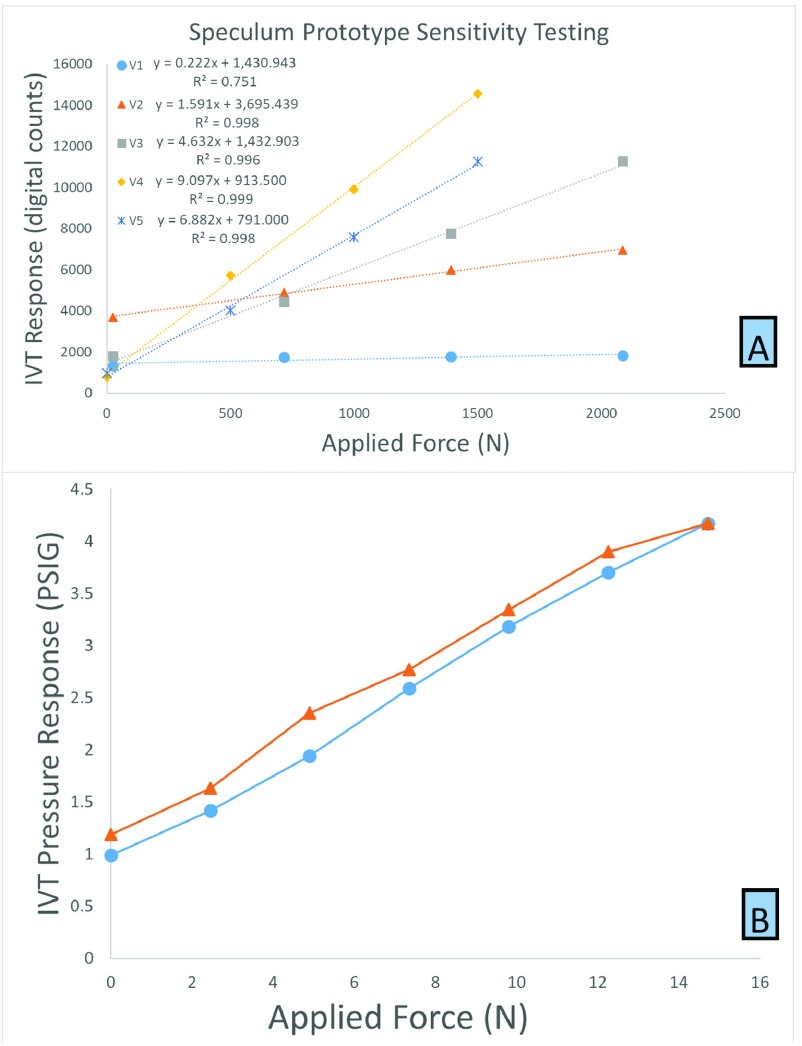
(A) Response linearity of the prototype versions shown in Figure 3A. (B) Response during loading and unloading of the stainless steel removable sensor speculum. Circles are the response during increasing loads and triangles are the response during decreasing loads.

The lower static bill of the speculum was found to be the most complicated part to cast and became the limiting part for the number of speculums produced. This was because five of the lower static bill casts had thin wall defects from the investment process, and another four lower static bill parts had to be discarded due to drill drift or fractured drill bits in the machining operations. After press fitting linkage pins 10 speculums had acceptable parallelism between the two bills for clinical use, all of which were tested for linearity with at least one IVT. A representative loading and unloading response curve of the IVT when placed in the speculum is shown in Figure 5. The response curve shows slight hysteresis, which is to be expected in a mechanically-coupled system such as the four bar linkage used in our design.

The coefficient of determination (R^2^) for each pairing in the inter/intra device benchtop study is shown in Table 1. The high linearity with all pairings was important for our study, as selective pairing of an IVT to a speculum would not be feasible for the study throughput nearing 20 participants per week. This way our coordinators could select any sensor and any speculum for a visit without worry of systematic error.

**TABLE 1:** Inter-Intra Speculum Linearity Test

R^2^	Sensor 1	Sensor 2	Sensor 3	Sensor 4	Sensor 5
Speculum 1	0.997	0.982	0.989	0.987	0.990
Speculum 2	0.994	0.993	0.993	0.972	0.992
Speculum 3	0.980	0.998	0.995	0.983	0.987
Speculum 4	0.992	0.983	0.996	0.985	0.979
Speculum 5	0.996	0.988	0.999	0.982	0.984

The final system verification results are shown in Table 2. The forces applied by hanging masses were compared with the software reported values, which showthe design requriements of accuracy within 5% full scale is satisfied. This verified the calibration and activity calculation procedures in the app functioned properly and produced accurate results.

**TABLE 2:** Final Device Bench Testing Using Entire System of Devices and Software

Events			Air	Calibrat	Valsalva	Rest 1	Kege Hold	Rest 2	Kege Rep	Rest 3	Air 2
Mass (g)			0	Spring Clamp	200	0	500	0	1000	0	0
Force (N)			0	10.40	1.96	0	4.90	0	9.81	0	0
Speculum Reponse (N)	IVT Number	715	−0.02	10.62	2.00	0.37	5.13	0.37	9.93	0.18	0.02
		705	−0.01	10.32	2.05	0.36	5.14	0.05	9.81	0.15	0.12
		714	−0.01	10.36	2.17	−0.04	5.24	0.02	9.94	0.03	0.00
		711	−0.03	10.31	2.09	0.03	5.63	0.04	10.16	0.07	0.01
		707	−0.02	10.36	1.75	0.03	4.83	0.08	9.93	0.05	0.03
		708	0	10.30	2.15	0.03	5.07	−0.01	9.65	0.20	0.11
	Average		−0.02	10.38	2.04	0.13	5.17	0.09	9.90	0.11	0.05
	Std. Dev.		0.01	0.12	0.15	0.18	0.26	0.14	0.17	0.07	0.05

**FIGURE 6.**
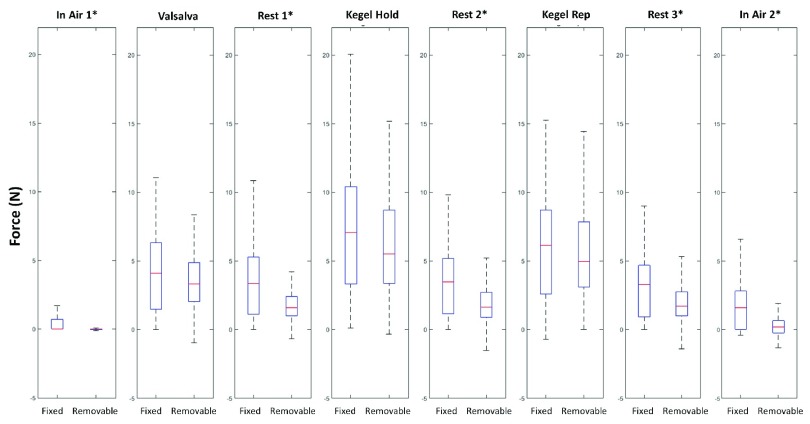
Comparison of data collected with the Fixed-bill (N = 85) and Removable Sensor (N = 165) Force Measuring Speculums. * denotes statistically significant difference in measurements (two-tailed t-test, unequal variance, alpha =.05).

The clinical measurements obtained with the removable sensor speculum were compared to the clinical measurements obtained using the fixed-bill speculum and showed good agreement (Figure 6). These measurements represent the same study population (MAP study) and are all measured in primiparous women at 1 year postpartum [16].

## IV. Discussion

The newly designed removable sensor speculum utilizes a novel design feature of a replaceable sensor that combines with the patient-contacting portion of the speculum. This allows the patient-contacting portion to undergo the rigorous sterilization reprocessing process required for reuse in a clinical environment while the single-use sensor can be used and simply discarded.

The sterilization requirements of the speculum drove the decision to use 316 stainless steel which is typically not used in prototyping or small production due to specialty tooling required for manufacture. But consultation with an experienced machinist helped us to develop the production method for rapid prototyping with this stainless steel. An aluminum speculum was much easier to machine and provided the precision master piece to cast the reproduction silicone molds. These molds produced highly accurate investment waxes that were cast into stainless steel without any specialty tooling. The cast pieces were then hand polished and assembled with simple drilling and pressing operations to produce the final speculums. This process significantly reduced time and cost to manufacture a moderately complex stainless steel device.

The electronic design of the equipment follows IEC/ISO 60601-1 guidance for a BF type device. The power is an isolated low voltage dc source and the IVT is made of insulating silicone with a double insulated wire that connects to the IM. The electrical lines connecting the IVT and the IM are protected from electrostatic shock using low capacitance TVS arrays, and overcurrent in the case of a short or open charged line with a power management IC with thermal regulator, series resistors on external lines, and software programmed MOSFET switches. All these safety features are located within the IM so that only low current is passed to the applied part of the device. The only change made to the electronics for this design was the addition of the BLE 113 module. The BLE113 Bluetooth chip added to the circuit complies with FCC and CE regulations for radiation exposure. Further testing of the device would be performed if it were to become a marketed device.

The use of a companion app was required for visual feedback and correct placement of the speculum in the vagina [7]. In addition to displaying VCF, we used the app to calculate unique IVT/speculum pair calibrations, activity prompts for the user, and automatic calculation of results. The spring clamp provided a simple and quick method of calibration just before use. Overall, the MAP study specific software was well received by our study coordinators and streamlined VCF data collection for the large scale clinical study.

The variations in measurement offset and response slope between IVTs and speculums is likely associated with variations in IVT placement and the resulting contact area of the dynamic upper bill affecting the force-to-pressure transfer function sensitivity. Calibration of each IVT/speculum pair immediately before use not only increased measurement accuracy, but also made for an easy to use system for our coordinators who could select any IVT and speculum when conducting a visit. The mass of the upper dynamic bill is supported by the IVT, and force measurements can be affected by the angle of the device. Ptaszkowski *et al.* [19] found that functional EMG activity was slightly higher when women who were standing contracted the pelvic floor muscles in posterior tilt compared to anterior tilt position. While we attempted to reduce variability in positioning by taking all measurements in a standardized fashion in the lithotomy position, it is possible that pelvic tilt may have influenced individual force measures.

The measurement differences between the two versions of speculum arose during the in air and the resting measurements (Figure 6). A pair-wise comparison of the same participants on both instruments would be an ideal comparison to show similarity or not, but it was not practically achievable in this study. Given relative large sample size of the study, assuming random sampling from two study populations, the conditional differences derived from t test were equivalent to the marginal differences (and the true differences), if there were any. Since the t test shows non-significant differences in some measures (e.g. the more important measures of Valsalva, Kegel Hold, and Kegel Rep), it was a good indication of similarity between the two populations. We believe they are likely due to the different design principles and software. For example, the fixed sensor speculum did not report values below 0 N due to software truncation. Because in-air measurements should be normally distributed around 0 N, the statistical difference between the two speculums for the in air measurements is likely due to censoring of the negative values to 0 N by the fixed sensor speculum software. As for the resting measurements, the statistical differences can have arisen from the differing measurement principles between the two designs. The shear-beam design of the fixed sensor speculum and the parallel bar linkage design of the removable sensor speculum transfer forces to their sensors very differently. In the case of the fixed sensor speculum, a force applied to the bill is read as a (different) bending moment at the two strain gages on the bill from which the (shear) force is calculated because of the known separation distance between the gages. For the removable sensor speculum, the same forces applied distally and proximally register very similar measurements. One difference between the shear-beam design and parallel bar linkage system is the greater excursion involved in the second design which was always less than 3 mm under the 10.4 N test load. Whether or not this influences readings will require further investigation. However, from a clinical point of view, the primary force variable chosen a priori in our research study, “kegel rep” (that is, the average force of three consecutive forceful pelvic floor muscle contractions) does not differ significantly between the two groups tested with the two different speculums. Consistent with previous research [6], [7], [20], the value of “in Air 1” is subtracted from “kegel rep” to obtain the final analytic variable, which is also similar between groups.

The removable sensor speculum was designed for the sole purpose of obtaining VCF measurements in an ongoing large scale clinical study. The use of the single-use IVT as the sensing element significantly reduced the design cycle time, taking approximately only 4 months from initial design to device delivery and routine usage. The design inputs for this device mandated that a sterile speculum be used for each clinical study participant. This requirement was met by using the existing IVT in a novel way that allowed for both accurate measurements of VCF and IAP in the same visit with little additional cost, approximately $4 USD per participant, to the study. This same design principle could be applied to a future, next-generation design which incorporates a removable sensor that does not contact the patient and a stainless steel speculum that can be easily resterilized by standard reprocessing techniques. A removable sensor design where both the sensor and speculum are reusable may be economical such that it could be routinely used during pelvic exams and replace the less objective Brinks and Oxford tests.

## V. Conclusion

A single-use intra-abdominal pressure sensor was successfully fitted to a custom speculum in order to also collect vaginal closure force data in a large-scale clinical trial. Dual purpose utilization of the pressure sensor allowed for rapid integration into the clinical study. The removable sensor speculum was coupled with a wireless data transmission system to provide real time data visualization to facilitate proper speculum placement and data storage. Clinical measurements were similar to the improved vaginal speculum described previously [6].

## Conflict of Interest

The Authors declare that there is no conflict of interest.
